# No-go trials in task switching: effects on the task-set and task-space level

**DOI:** 10.1007/s00426-021-01566-7

**Published:** 2021-07-31

**Authors:** Juliane Scheil, Thomas Kleinsorge

**Affiliations:** Leibniz Research Centre for Working Environment and Human Factors, Ardeystraße 67, 44139 Dortmund, Germany

## Abstract

A common marker for inhibition processes in task switching are *n* − 2 repetition costs. The present study aimed at elucidating effects of no-go trials on *n* − 2 repetition costs. In contrast to the previous studies, no-go trials were associated with only one of the three tasks in the present two experiments. High *n* − 2 repetition costs occurred if the no-go task had to be executed in trial *n* − 2, irrespective of whether a response had to be withheld or not. In contrast, no *n* − 2 repetition costs were visible if the other two tasks were relevant in *n* − 2. Whereas this *n* − 2 effect was unaffected by whether participants could reliably exclude a no-go trial or not, effects of no-gos in trial *n* were determined by this knowledge. The results differ from effects of no-go trials that are not bound to a specific task. It is assumed that the present no-go variation exerted its effect not on the response level, but on the level of task sets, resulting in enhanced salience of the no-go task that leads to higher activation and, as a consequence, to stronger inhibition. The dissociation of the effects on no-gos in trials *n* − 2 and *n* as a function of foreknowledge suggests that the balance between activation and inhibition is shifted not only for single trials and tasks, but for the whole task space.

## Introduction

In everyday life, humans are constantly confronted with the necessity to adapt their behavior according to environmental constraints. This flexible control of behavior has been in the focus of cognitive psychology research for the past decades. Under lab conditions, the task switching paradigm is a frequently used tool to investigate how humans switch among different goals and actions (cf. Kiesel et al., 2010, for a review).

One of the mechanisms that is considered to play a key role in switching from the performance of one task to the performance of another task is the inhibition of the cognitive processes that the switched-to task differs from the switched-from task. In principle, this inhibition can apply to the whole cognitive configuration of the switch-from task (called the ‘task set’, cf. Monsell, 2003), or it can apply to more specific aspects as individual stimulus–response mappings, the motor part of the task set, or the response that was most recently emitted.

A common measure for inhibitory processes during task switching is the so-called *n* − 2 repetition costs (Mayr & Keele, 2000). These costs can be observed when switching among at least three different tasks A, B, and C, and manifest as higher reaction times and error rates in sequences of type ABA (i.e., when the task in the current trial equals the task in trial *n* − 2) as compared to CBA sequences (i.e., an *n* − 2 nonrepetition). These costs are explained by inhibitory processes aimed at reducing interference when switching among different tasks. In each trial, the cognitive representation of the relevant task, the task set or part of it, becomes inhibited after response execution (e.g., Mayr, 2002). This leads to costs when the same task set has to be activated again soon after, as it is the case in ABA sequences. In CBA sequences, on the contrary, the last encounter with task A lies at least three trials back, minimizing aftereffects of inhibition.

Although there has been plenty of research focusing on *n* − 2 repetition costs during the last 20 years, it is still under debate how the inhibition process underlying this phenomenon can be characterized. On the one hand, it is possible that some kind of self-inhibitory process is triggered at the end of each trial, automatically inhibiting the task set when a response is executed (Mayr & Keele, 2000). However, there are arguments against such a nonflexible process. Most importantly, *n* − 2 repetition costs vary in their size and presence depending on aspects of not only the inhibited task but the whole task environment, like task dominance (Jost et al., 2017). Furthermore, *n* − 2 repetition costs are still present when task repetitions are included in the trial sequence, a condition in which a self-inhibition process would hamper performance (see also Grange et al., 2013, for a discussion). Besides this self-inhibition account, a lateral inhibition process has been proposed, assuming that during the preparation of one task, inhibition is spread to the other tasks to minimize interference. This principle was, for example, taken up in the computational model of Sexton and Cooper (2017). In this model, each task is represented by a task demand unit that has a certain activation level. If two of these task demand units have activation levels larger than zero, the resulting conflict is detected by so-called conflict monitoring units. As a consequence, these units cause the inhibition of the currently irrelevant task demand units to reduce interference.

*N* − 2 repetition costs have been observed under different experimental conditions. However, many characteristics of the processes underlying this effect are still unknown (see Koch et al., 2010, for a review). It has been shown that the occurrence and size of *n* − 2 repetition costs are affected by characteristics of the tasks as well as the whole task environment. Among other factors, response execution was shown to modulate this effect. For example, Schuch and Koch (2003) introduced no-go trials in their experiments. *N* − 2 repetition costs were absent when no response had to be executed in trial *n* − 1. The authors concluded that the inhibition process underlying *n* − 2 repetition costs is triggered by response execution, while response preparation alone is not sufficient to produce the costs (but see Kleinsorge & Gajewski, 2004). Other response-related factors, like the overlap of response sets, have an influence on *n* − 2 repetition costs as well (Gade & Koch, 2007). Furthermore, *n* − 2 repetition costs were shown to be affected by the mental execution of a response (i.e., motor imagery, Scheil et al., 2020). These findings suggest a rather late (i.e., motor) locus of inhibition. However, not all variations of response-related factors have an influence on inhibition in task switching. For example, Scheil (2016) found no modulation of *n* − 2 repetition costs by changing the stimulus–response mapping of one of the tasks in the course of the experiment, an effect that was certainly to expect if the inhibition process underlying *n* − 2 repetition costs targets at the level of response execution alone as disrupting the association of a particular stimulus–response mapping should have affected the ease of selecting the respective response. Therefore, more research is needed to better understand how response-related manipulations in task switching experiments exert their influence on *n* − 2 repetition costs.

The present study aimed at further investigating effects of no-go trials on *n* − 2 repetition costs. In contrast to Schuch and Koch (2003), who focused on effects on *n* − 2 repetition costs associated with response selection, the focus of our study lies on the additional (motor) inhibition process that is triggered by withholding a response. The main difference between these two accounts is that Schuch and Koch (2003) centered around effects of inhibiting a response per se, while we intend to manipulate the inhibition process directly and look for aftereffects of inhibition in terms of a modulation of *n* − 2 repetition costs. Therefore, the main analyses relate to the trial type in trial *n* − 2 instead of trial *n* − 1, as it was the case in Schuch and Koch’s study. Our hypotheses are based on the assumption of Scheil and Kleinsorge (2014) that each task is activated in trial *n* − 2 and inhibited during the preparation interval of trial *n* − 1. Specifically, we hypothesize that the additional need of inhibition in no-go trials at *n* − 2 affects the size of *n* − 2 repetition costs. Regarding the direction of this effect, two possibilities arise. On the one hand, *n* − 2 repetition costs may vanish if a no-go is presented in trial *n* − 2 because the inhibition of response-related parts of the task set reduces the need for further inhibition. This is similar to Schuch and Koch’s reasoning that a no-go signal prevents a task set from becoming fully activated. On the other hand, it is possible that *n* − 2 repetition costs inflate because the task set is inhibited twice (due to motor inhibition in *n* − 2 and due to task set inhibition in *n* − 1). To relate these two possibilities to the model of Sexton and Cooper (2017), we hypothesize that a no-go in trial *n* − 2 either decreases the activation level of the respective task demand unit, minimizing its potential interference and, therefore, subsequently reduce the inhibition triggered by the conflict monitoring unit, leading to smaller *n* − 2 repetition costs. On the other hand, an additional motor inhibition demand that is specifically tied to one of the tasks may increase the activation level of the respective task demand unit, causing higher interference during the next trial, which leads to more inhibition triggered by the conflict monitoring unit and, consequently, to higher *n* − 2 repetition costs.

In contrast to the study of Schuch and Koch (2003), no-go trials were presented for only one of the three tasks. By doing this, we wanted to observe whether possible effects are selectively visible for sequences involving no-go trials, or whether effects are of a more global nature, e.g., by spreading to all trials of the no-go task, or by spreading to all tasks.

## Experiment I

### Method

#### Participants

30 subjects participated. One subject had to be discarded due to not following the instructions. The final sample consisted of 29 subjects (4 male) with a mean age of 22.8 years (range 19–29). All had normal or corrected-to-normal vision. To estimate the achieved power of the relevant effect, the interaction of Task Sequence and lag2_No-Go, we decided to use the results of Schuch and Koch’s (2003) second experiment to estimate the effect size, because this experiment equals the present one most closely. As no effect size was given in this study, it was calculated based on the *F* statistics of the interaction (Task Sequence × No-Go) in RT data of Experiment 2. This yielded a partial eta square of .468. Using MorePower 6.0 (Campbell & Thompson, 2012), the estimated power is .99 for this sample size.

#### Stimuli, tasks, and apparatus

Stimuli consisted of two different shapes (x and +) presented in yellow or blue and with a size of either 3 cm × 3 cm or 6 cm × 6 cm. Task cues consisted of a dark grey diamond, square, or triangle surrounding the position of the imperative stimulus with a size of about 7 cm × 7 cm. Participants switched among three perceptual decision tasks in which they had to judge the stimuli regarding their size (large vs. small, indicated by the diamond), color (yellow or blue, indicated by the square), or their shape (x or + , indicated by the triangle). Furthermore, an auditive go or no-go signal was presented 100 ms after the presentation of the imperative stimulus. This signal consisted of a high tone for no-go trials and a low tone for go trials. Two of the tasks were always presented together with the go signal (go tasks). For the third task, 50% of all trials were no-go trials (no-go task). Which of the task constituted the no-go condition was varied between subjects. As no task repetitions were allowed in this experiment, the same holds for repetitions of no-go trials.

All tasks occurred with equal frequency. Direct stimulus repetitions were not allowed. Stimuli were presented centrally on a light-grey background. Viewing distance was not controlled but approximated 60 cm. Participants pressed the ‘y’-key of a German QWERTZ keyboard for small, blue, and x-shaped stimuli and the ‘-‘-key for big, yellow, and +-shaped stimuli.

#### Design and procedure

After giving informed consent, participants were provided with onscreen instructions in which the tasks and the meaning of the task cues were explained. Instructions emphasized speed as well as accuracy. The experiment was run in a single session that took about 70 min. It started with a practice block of 72 trials. In this block, that did not contain no-go trials, all tasks were practiced separately for 14 trials each. After that, 30 mixed trials without task repetitions followed. The test session consisted of 18 experimental blocks of 72 trials each.

Each trial began with the presentation of a fixation mark for 600 ms. Then the task cue was presented for 1000 ms. After that, the cue disappeared and the imperative stimulus was presented for 2500 ms or until the participant’s response. 100 ms after the onset of the imperative stimulus, the go or no-go tone was presented for 50 ms. In case of an error, error feedback was presented for additional 1000 ms; in case of RTs slower than the RT deadline of 2500 ms, RT feedback was presented for additional 1000 ms.

### Results

The practice block was not analyzed. Furthermore, the first three trials of each block were excluded, as were sequences involving an error or a missing response in trials *n* − 2 or *n* − 1. Furthermore, only performance of go trials could be analyzed, as no reaction was given in no-go trials. From RT data, errors in the current trial were also excluded.

For ER data, two kinds of errors were possible, pressing any key in a no-go trial or pressing the wrong key in a go trial. The mean error proportions were 6.0% for no-go trials and 5.1% for go trials.

In this experiment, two kinds of tasks were presented. On the one hand, one task that was possibly associated with no-go trials, which in the following is labeled no-go task. On the other hand, two tasks for which no-go trials were excluded, which are labeled go-tasks. Furthermore, two kinds of trials were possible, go trials on the one hand and no-go trials on the other hand. For the go-tasks, only go trials were present. This condition is labeled “go/go-task”. For the no-go task both trial types were present. Consequently, the two conditions are labeled “no-go/no-go task” if a no-go trial was present and “go/no-go task” for go trials.

Importantly, as task repetitions were not allowed, no-go trials could not be present at both position *n* − 1 and *n* − 2. The only possibility for two no-go trials within a sequence are ABA sequences in which task A is the no-go task. However, in these cases, sequences with a no-go trial at the serial position not considered in the respective analyses were excluded.

#### Go condition in trial *n* − 2

RT and ER data of go trials were analyzed using a repeated measures ANOVA with the within-subjects factors Task Sequence (CBA vs. ABA), and lag2_No-Go (identity of the condition in trial *n* − 2: no-go/no-go task, go/no-go task, go/go task).

**Fig. 1:**
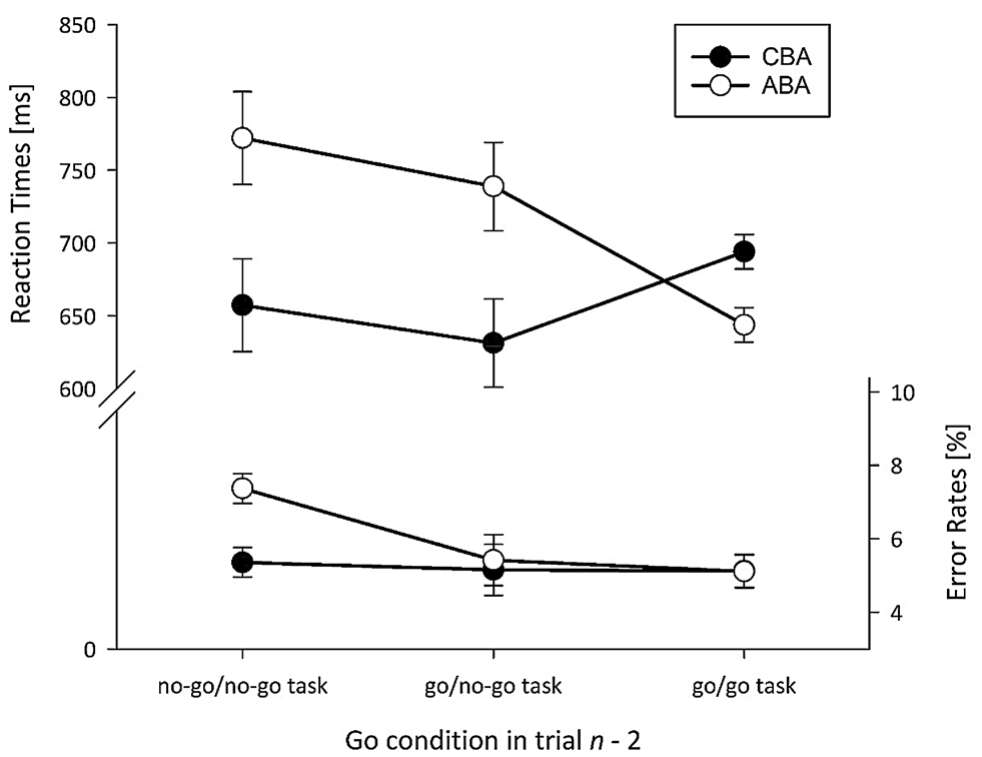
Experiment I: Mean RT [ms] and ER [%] as a function of Go condition in trial *n* − 2 and Task Sequence. Error bars represent the *SE* of paired ABA/CBA differences (cf. Pfister & Janczyk, 2013)

For RT data, a significant main effect of Task Sequence occurred, *F* (1, 28) = 12.09, *p* = .002, $\eta_{{\text{p}}}^{2}$ = .30, due to a mean *n* − 2 repetition cost of 57 ms. The main effect of lag2_No-Go was significant as well, *F* (2, 56) = 10.98, *p* < .001, $\eta_{{\text{p}}}^{2}$ = .28. RTs for sequences with a no-go in trial *n* − 2 were significantly higher (715 ms) compared to go trials of the no-go task (685 ms, *p* = .004, Newman–Keuls corrected) and go trials of the go tasks (669 ms, *p* < .001), while the latter two did not differ (*p* = .107). Both factors interacted, *F* (2, 56) = 13.76, *p* < .001, $\eta_{{\text{p}}}^{2}$ = .33 (cf. Fig. 1): Significant *n* − 2 repetition costs were visible for no-go trials (115 ms, *p* < .001) and go trials of the no-go task (108 ms, *p* < .001), while for go trials of the go tasks, a nonsignificant *n* − 2 repetition benefit of 50 ms occurred (*p* = .121, 95%-CI 74.59–25.91 ms).

For ER data, the main effect of Task Sequence was not significant, *F* (1, 28) = 1.49, *p* = .232. In the ‘standard condition’ with the go task in trials *n* − 2 and *n*, the usual significant *n* − 2 repetition cost was reversed into a (nonsignificant) benefit (95%-CI 3.6–1.0%). The main effect of lag2_No-Go was significant, *F* (2, 56) = 3.37, *p* = .041, $\eta_{{\text{p}}}^{2}$ = .11. ERs for sequences with a no-go in trial *n* − 2 were significantly higher (6.4%) than ERs of go trials of the no-go task (5.3%, *p* = .043, Newman–Keuls corrected) and marginally higher than the go trials of the go tasks (5.1%, *p* = .051), while the latter two did not significantly differ (*p* = .747). The interaction of both factors was not significant, *F* (2, 56) = 1.32, *p* = .276.

#### Go condition in trial *n* − 1

In addition to our main analyses, we investigated whether the go condition in trial *n* − 1 had an effect on main RT and ER data and on *n* − 2 repetition costs. For this purpose, RT and ER data were analyzed using a repeated measures ANOVA with the within-subjects factors Task Sequence (CBA vs. ABA), and lag_No-Go (identity of the condition in trial *n* − 1: no-go/no-go task, go/no-go task, go/go task).

For RT data, the main effect of lag_No-Go was significant, *F* (2, 56) = 8.86, *p* < .001, $\eta_{{\text{p}}}^{2}$ = .24. RTs were significantly higher after no-go trials (710 ms, *p* = .001) than after go trials of either the no-go task (662 ms, *p* = .001) or the go tasks (666 ms), while there was no difference between the latter two (*p* = .735). The interaction with Task Sequence was not significant, *F* = 1.32, *p* = .275.

For ER data, none of the effects was significant, all *F*’s < .1, all *p*’s > .713.

#### Task in trial *n*

In addition, we checked whether participant’s performance was affected by the task identity in trial *n*, that is, whether the current task was the no-go task or one of the go tasks. For this purpose, RTs and ERs of go trials were analyzed using a repeated measures ANOVA with the within-subjects factors Task Sequence (CBA vs. ABA), and Task (no-go task vs. go task). For these analyses, no-go trials in *n* − 1 and *n* − 2 were excluded.

For RT data, the main effect of Task was significant, *F* (1, 28) = 13.62, *p* < .001, $\eta_{{\text{p}}}^{2}$ = .33. Participants responded significantly faster when the current task was a go task (645 ms) as compared to the no-go task (735 ms). The interaction with Task Sequence was not significant, *F* < 1, *p* = .583.

For ER data, none of the effects was significant, all *F*’s < 1, all *p*’s > .632.

For the analysis of main interest, involving the no-go condition in trial *n* − 2 as a factor, a significant interaction of no-go condition and Task Sequence was visible: *n* − 2 repetition costs were present when the no-go task was the relevant one in trial *n* − 2, irrespective of the actual trial type, that is, whether it was a go trial or a no-go trial. Thus, it seems that this effect is independent of potential motor inhibition due to the requirement to withhold a response. Instead, the enhanced need to inhibit the task that was active in trial *n* − 2, visible as high *n* − 2 repetition costs, seems to be bound to the no-go task itself. A possible explanation may be that the information about the no-go condition was incorporated in the task set, thereby affecting all trials as soon as the respective task set was activated.

The notion that task inhibition, manifested as *n* − 2 repetition costs, is triggered at the level of abstract task identity was supported by Regev and Meiran (2016, 2017). In their studies, they successfully disentangled two different kinds of inhibition active during task switching, namely task set inhibition (as the process underlying *n* − 2 repetition costs) on the one hand and competitor rule suppression on the other hand. Competitor rule suppression is characterized as an inhibition process that selectively targets response rules that have been activated in the previous trial and that are incongruent with the response rule in the current trial; thereby, causing interference. More precisely, CRS+ trials (which are trials that are affected by the kind of response conflict triggering CRS) are characterized by the need to execute a response that has been incongruent during trial *n* − 1 and, therefore, had to be suppresses during trial *n* − 1, irrespective of whether the response per se switches or repeats. As a consequence, competitor rule suppression is assumed to target not a task set as a whole (which is supposed to be the case for the inhibition process underlying *n* − 2 repetition costs), but specific stimulus–response links. More precisely, the conceptual difference between inhibition and CRS is that *n* − 2 repetition costs reflect “the performance cost resulting from the suppression of a recently performed task rule, regardless if this rule has caused response competition. Conversely, CRS reflects performance cost due to suppression of a competitor rule regardless of whether this rule has been recently executed” (Regev & Meiran, 2016, p. 626). That is, both processes differ with regard to their target.

The fact that competitor rule suppression operates at the level of response rules raises the question whether the manipulation of no-go trials in the current experiment also affects this phenomenon. The investigation of effects of the no-go condition on competitor rule suppression may shed light into the way the no-go trials affect performance. Specifically, if the no-go condition was really bound to the task set in the present case, no effect on competitor rule suppression is assumed to be visible. In contrast, if the no-go condition exerts its influence on the level of response preparation or response execution, it should also affect competitor rule suppression. Therefore, we ran additional analyses to investigate effects of the no-go condition on competitor rule suppression. These analyses were conducted following Regev and Meiran’s (2016, 2017) way of data presentation. In a first step, two preliminary analyses were run on RT and ER data to ensure that the necessary preconditions for CRS effects to be observed are met. The first analysis is suited to ensure that the CRS effect is not affected by other kinds of conflict effects. More specifically, there are trials that cannot be defined as CRS+ trials but may also reflect some kind of postconflict effect. This is the case when the stimulus–response mappings of trial *n* − 1 do not bear a conflict with regard to the task which is relevant in trial *n*, but with regard to the third task. The second preliminary analysis was run to ensure that there is a task rule congruency effect present, because this is a necessary precondition for interference effects due to incongruent task rules. Only when (1) no other postconflict effects affect the data pattern and (2) a task rule congruency effect is present, the results regarding CRS effect can be interpreted.

#### Preliminary analyses

In a first step, we checked whether conflict trials in *n* − 1 that cannot be defined as CRS+ affected the data pattern. For this purpose, CRS+ trials were excluded from analyses, while CRS− trials remained. This includes partially incongruent trials (in which the mappings of trial *n* − 1 and trial *n* were congruent but the stimulus of trial *n* − 1 was incongruent with relation to the third potential task) as well as fully congruent trials (with stimuli whose features would afford the same response for all three tasks). Then, RT and ER data were subjected to repeated measures ANOVAs with the within-subjects factors lag_Congruency (congruent vs. incongruent) and lag_No-Go (identity of the condition in trial *n* − 1: no-go/no-go task, go/no-go task, go/go task). For RT data, neither the main effect of lag_Congruency nor its interaction with lag_No-Go were significant (*F* < 1, *p* = .937 and *F* = 1.96, *p* = .150, respectively). The same holds for ER data: Neither the main effect of lag_Congruency nor its interaction with lag_No-Go were significant (*F* < 1, *p* = .350 and *F* < 1, *p* = .872, respectively).

In a second step, we examined whether a task rule congruency effect, which is assumed to be critical for CRS to take place, exists in our data. For this purpose, RT and ER data were subjected to repeated measures ANOVAs with the within-subject factor Congruency (congruent, mixed, incongruent). For RT data, the effect of Congruency was significant, *F* (2, 56) = 5.81, *p* = .005, $\eta_{{\text{p}}}^{2}$ = .17. RTs were highest for incongruent stimuli (705 ms), of intermediate size for mixed stimuli (700 ms), and lowest for congruent ones (684 ms). For ER data the effect of Congruency was significant as well, *F* (2, 56) = 23.26, *p* < .001, $\eta_{{\text{p}}}^{2}$ = 0.45. ERs were highest for incongruent stimuli (8.3%), of intermediate size for mixed stimuli (4.9%), and lowest for congruent ones (3.0%).

#### Main analyses

To investigate effects of CRS, RT and ER data were subjected to repeated measures ANOVAs with the within-subjects factors CRS (CRS+ vs. CRS−) and lag_No-Go (identity of the condition in trial *n* − 1: no-go/no-go task, go/no-go task, go/go task).

For RT data, the main effect of lag_No-Go was significant, *F* (2, 56) = 9.28, *p* < .001, $\eta_{{\text{p}}}^{2}$ = .25, because RTs were higher after no-go trials (710 ms) compared to go trials of either the no-go task (663 ms) or the go tasks (665 ms). The main effect of CRS was significant as well, *F* (1, 28) = 5.61, *p* = .024, $\eta_{{\text{p}}}^{2}$ = .17, because the CRS+ condition was associated with higher RTs (685 ms) compared to the CRS− condition (674 ms). Importantly, the CRS effect was not modulated by lag_No-Go, *F* (2, 56) = 1.55, *p* = .219.

For ER data, the main effect of lag-No-Go was not significant (*F* < 1, *p* = .742). The main effect of CRS was marginally significant, *F* (1, 28) = 2.93, *p* = .098, $\eta_{{\text{p}}}^{2}$ = .09, because ERs of the CRS+ condition tended to be higher (5.4%) than those of the CRS− condition (4.7%). The interaction of both factors was not significant (*F* < 1, *p* = .740).

### Discussion

In Experiment I, the presence of no-go trials significantly affected *n* − 2 repetition costs. Large *n* − 2 repetition costs were visible after the no-go task was the relevant one in *n* − 2, while they were absent for the go tasks. Moreover, the fact that go trials of the no-go task show *n* − 2 repetition costs equal to those of actual no-go trials suggests that effects are not selectively bound to no-go trials but spread to all trials associated with the potential no-go task. This further indicates that the effect is not due to motor inhibition in no-go trials directly affecting the size of *n* − 2 repetition costs, because in this case, go trials of the no-go task should show the same data pattern as trials of the go tasks. Instead, it seems that the occurrence of no-go trials, at least when bound to a specific task, shows effects not only on the response level but also on the level of task sets. This is also supported by the effect of task type in trial *n*: Participants react significantly slower when the no-go task has to be executed, suggesting that as soon as the task cue indicated the task associated with no-gos, they either withhold their response or prepare for it to a smaller degree as compared to tasks that are not associated with no-go trials.

To further investigate the assumption that the no-go condition exerted its influence on the level of task sets, a second series of analyses was run to investigate effects of the no-go condition on competitor rule suppression. Although the significant main effect, at least in RT data, indicates that competitor rule suppression took place, this effect was not modulated by the no-go condition. This further supports the notion that no-go trials did not just affect response-related processes, as it would have been assumed if the no-go condition was not bound to a specific task. Instead, it seems that being associated with the possibility of a no-go became part of the task set, thereby affecting task set inhibition and causing high *n* − 2 repetition costs. What can be ruled out on the basis of these observations is that response inhibition per se reduces the activation of a task to a degree that prevents inhibition to occur.

An additional note should be made regarding possible effects of episodic retrieval (cf. Grange et al., 2017). It has been argued before that *n* − 2 repetition costs are, at least partially, due to episodic retrieval that leads to partial mismatches in ABA sequences but not in CBA sequences and, therefore, inflates effects due to task set inhibition. However, in the present experiment, episodic retrieval should have caused higher *n* − 2 repetition costs only after actual no-go trials in *n* − 2, because in this case, the response may be tagged with a “do not execute” label that is retrieved during the next encounter with the same task, which is trial *n* in ABA sequences. However, the fact that all trial types of the no-go task are affected to the same degree speaks against the notion of episodic retrieval guiding the data pattern of the present experiment.^1^

In contrast to the study of Schuch and Koch (2003), no-go trials in *n* − 1 did not significantly affect *n* − 2 repetition costs. However, whereas participants could to some degree predict the occurrence of a no-go trial in the present study, this was not the case in the experiments of Schuch and Koch, because no-go trials were possible for all of the three tasks. As a consequence, one may assume that no-go effects were bound to the response level, while a possible need for motor inhibition could be embedded in the task set in the present study.

Importantly, participants were not explicitly told that no-go trials occurred only for one of the tasks. However, being asked after the experiment, all of them reported to have noticed it. As a consequence, the question arises whether the data pattern of Experiment I is due to participants’ purposefully adapting their response to the no-go task, and whether this is true not only for the main effect on RTs but also for the effect on *n* − 2 repetition costs. Therefore, a second experiment was conducted in which we manipulated participants’ expectancy of no-go trials. No-go trials were validly cued by a slightly changed task cue. The same cue was used for a proportion of go trials of the no-go task (in the following called potential no-go trials) while the rest of these go trials were cued by the “usual” task cue. If participants change their response behavior on purpose, this should be only visible for potential no-go trials but not for the go trials of the no-go task, because the valid cue excludes a no-go in the latter condition.

## Experiment II

### Method

#### Participants

30 subjects participated (2 male) with a mean age of 22.8 years (range 18–29) participated. All had normal or corrected-to-normal vision. Considering the effect size of the Task Sequence × lag2_No-Go interaction that was found in Experiment I, this sample size also yields a power estimation of .99.

#### Stimuli, tasks, and apparatus

These were largely identical to Experiment I. The only difference concerned the no-go task. For this task, 33% of the trials were go trials with the task cue presented in dark grey, as it was the case for the other two tasks. In the remaining trials, the task cue was colored light grey. Participants were validly informed that a dark cue always indicated a go trial while a light cue might or might not indicate a no-go trial. This was the case in 50% of the trials with light grey cue (33% of all trials of the no-go task).

#### Design and procedure

This was identical to Experiment I.

### Results

The practice block was not analyzed. Furthermore, the first three trials of each block were excluded, as were sequences involving an error in trials *n* − 2 or *n* − 1. Furthermore, only performance of go trials could be analyzed, as no reaction was given in no-go trials. From RT data, errors in the current trial were also excluded. The mean error proportions were 4.9% for no-go trials and 5.3% for go trials.

#### *N* − 2 repetition costs

##### Go condition in trial *n* − 2

RT and ER data of go trials were analyzed using a repeated measures ANOVA with the within-subjects factors Task Sequence (CBA vs. ABA), and lag2_No-Go (identity of the condition in trial *n* − 2: no-go/no-go task, potential no-go (but actually go)/no-go task, go/no-go task, go/go task).

**Fig. 2:**
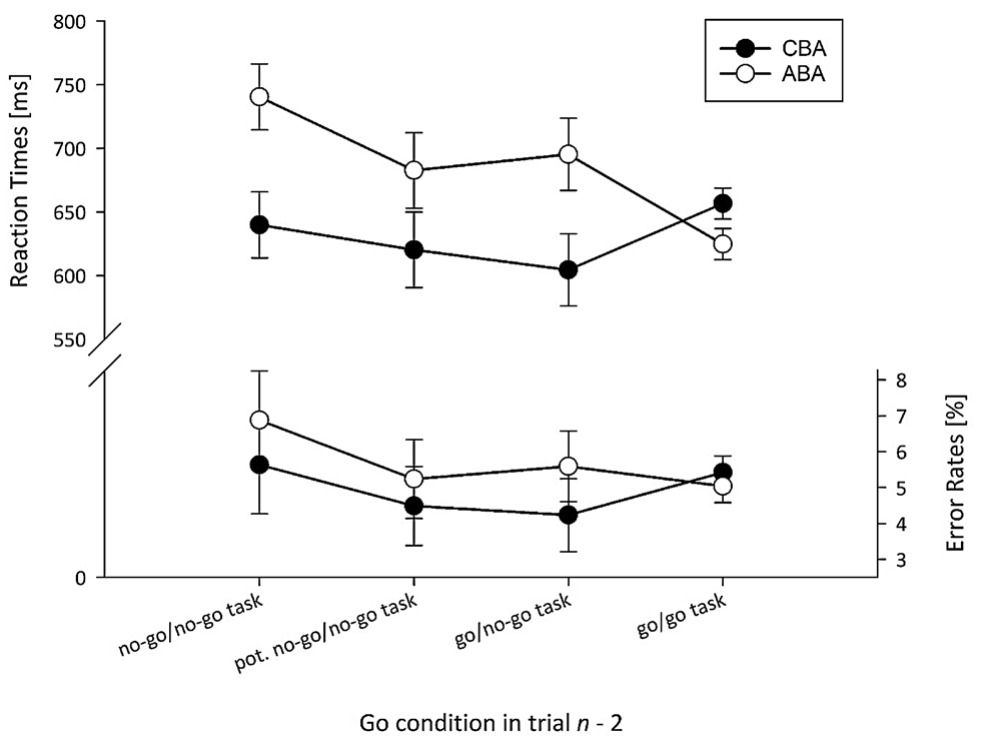
Experiment II: Mean RT [ms] and ER [%] as a function of Go condition in trial *n* − 2 and Task Sequence. Error bars represent the *SE* of paired ABA/CBA differences

For RT data, a significant main effect of Task Sequence occurred, *F* (1, 29) = 10.68, *p* = .002, $\eta_{{\text{p}}}^{2}$ = .27, due to a mean *n* − 2 repetition cost of 56 ms. The main effect of lag2_No-Go was significant as well, *F* (3, 87) = 7.49, *p* < .001, $\eta_{{\text{p}}}^{2}$ = .21. RTs for sequences with a no-go in trial *n* − 2 were significantly higher (690 ms) as compared to all other conditions (all *p*’s < .002, Newman–Keuls corrected), while there were no significant differences among potential no-go trials of the no-go task (651 ms), go trials of the no-go task (650 ms), and go trials of the go tasks (641 ms, all *p*’s > .422). Both factors interacted, *F* (3, 87) = 8.12, *p* < .001, $\eta_{{\text{p}}}^{2}$ = .22: Significant *n* − 2 repetition costs were present for all conditions of the no-go task (101 ms for no-go trials, *p* < .001; 63 ms for potential no-go trials, *p* = .033; 91 ms for go trials, *p* = .001), while a non-significant (*p* = .294, 95%-CI 56.90–6.93 ms) *n* − 2 repetition benefit of 32 ms occurred for the go task (cf. Fig. 2).

For ER data, the main effect of Task Sequence was not significant, *F* (1, 29) = 1.64, *p* = .210, $\eta_{{\text{p}}}^{2}$ = .24. In the ‘standard condition’ with the go task in trials *n* − 2 and *n*, the usual significant *n* − 2 repetition cost was reversed into a (nonsignificant) benefit (95%-CI 2.1–0.2%). The main effect of lag2_No-Go was marginally significant, *F* (3, 87) = 2.32, *p* = .081, $\eta_{{\text{p}}}^{2}$ = .07. ERs for sequences with a no-go in trial *n* − 2 tended to be higher (6.3%) compared to potential no-go trials of the no-go task (4.9%), go trials of the no-go task (4.9%), and go trials of the go tasks (5.2%). The interaction of both factors was not significant, *F* (3, 87) = 0.78, *p* = .508.

##### Go condition in trial *n* − 1

As in Experiment I, we investigated whether the go condition in trial *n* − 1 had an effect on main RT and ER data and on *n* − 2 repetition costs. For this purpose, RT and ER data were analyzed using a repeated measures ANOVA with the within-subjects factors Task Sequence (CBA vs. ABA), and lag_No-Go (identity of the condition in trial *n* − 1: no-go/no-go task, potential no-go/no-go task, go/no-go task, go/go task).

For RT data, the main effect of lag_No-Go was significant, *F* (3, 87) = 11.27, *p* < .001, $\eta_{{\text{p}}}^{2}$ = .28. RTs were significantly (*p*’s < .001) higher after no-go trials (705 ms) than after potential no-go trials (648 ms), go trials of the no-go task (617 ms), or go trials of the go tasks (645 ms), while there was no difference between the latter three (*p*’s > .077). The interaction with Task Sequence was not significant, *F* = 2.32, *p* = .081.

For ER data, the main effect of lag_No-Go was not significant, *F* < 1, *p* = .585. However, the interaction with Task Sequence was significant, *F* (3, 87) = 3.25, *p* = .025, $\eta_{{\text{p}}}^{2}$ = .10. *N* − 2 repetition benefits were visible for no-go trials (1.2%) and potential no-go trials (1.3%), while *n* − 2 repetition costs of 1.5% occurred after go trials of the no-go task. After go trials of the go tasks, no cost or benefit occurred (0%).

##### Task in trial *n*

As in Experiment I, we checked whether participant’s performance was affected by the task identity in trial *n*, that is, whether the current task was the no-go task or one of the go tasks. For the no-go task, there was furthermore a difference between trials with a light grey cue that indicated a potential no-go, and trials with dark grey cue that excluded a no-go. For this purpose, RTs and ERs of go trials were analyzed using a repeated measures ANOVA with the within-subjects factors Task Sequence (CBA vs. ABA), and Task (no-go task(light), no-go task(dark), or go task). For these analyses, no-go trials in *n* − 1 and *n* − 2 were excluded.

For RT data, the main effect of Task was significant, *F* (2, 58) = 19.47, *p* < .001, $\eta_{{\text{p}}}^{2}$ = .40. Participants responded significantly faster (both *p*’s < .001) when the current task was either a go task (622 ms) or a darkly colored no-go task (635 ms) compared to a lightly colored no-go task that indicated a potential no-go (747 ms), while the former two conditions did not differ (*p* = .542). The interaction with Task Sequence was not significant, *F* < 1, *p* = .752.

For ER data, none of the effects was significant, all *F*’s < 1, all *p*’s > .771.

#### Competitor rule suppression

##### Preliminary analyses

As in Experiment I, we first checked whether conflict trials in *n* − 1 that cannot be defined as CRS+ affected the data pattern. For this purpose, CRS+ trials were excluded, and RT and ER data were subjected to repeated measures ANOVAs with the within-subjects factors lag_Congruency (congruent vs. incongruent) and lag_No-Go (identity of the condition in trial *n* − 1: no-go/no-go task, potential go/no-go task, go/no-go task, go/go task). For RT data, neither the main effect of lag_Congruency nor its interaction with lag_No-Go were significant (*F* = 1.83, *p* = .186 and *F* < 1, *p* = .591, respectively). For ER data as well, neither the main effect of lag_Congruency nor its interaction with lag_No-Go were significant (*F* = 2.65, *p* = .114 and *F* = 1.07, *p* = .363, respectively).

In a second step, the presence of a task rule congruency effect was checked. RT and ER data were subjected to repeated measures ANOVAs with the within-subject factors Congruency (congruent, mixed, incongruent). For RT data, the effect of Congruency was not significant, *F* < 1, *p* = .873. For ER data, the effect of Congruency was significant, *F* (2, 58) = 68.72, *p* < .001, $\eta_{{\text{p}}}^{2}$ = .70. ERs were highest for incongruent stimuli (8.4%), of intermediate size for congruent stimuli (5.5%), and lowest for mixed ones (3.0%).

##### Main analyses

To investigate effects of CRS, RT and ER data were subjected to repeated measures ANOVAs with the within-subjects factors CRS (CRS+ vs. CRS−) and lag_No-Go (identity of the condition in trial *n* − 1: no-go/no-go task, potential go/no-go task, go/no-go task, go/go task). It should be noted that no CRS effect is expected for RT analyses, because no task rule congruency effect was visible.

For RT data, the main effect of lag_No-Go was significant, *F* (3, 87) = 11.64, *p* < .001, $\eta_{{\text{p}}}^{2}$ = .29, because RTs were higher after no-go trials (702 ms) as compared to potential no-go trials (648 ms), and go trials of either the no-go task (616 ms) or the go tasks (650 ms). The main effect of CRS was not significant, *F* < 1, *p* = .935. The interaction of both factors was not significant, *F* < 1, *p* = .785.

For ER data, the main effect of Congruency was not significant (*F* < 1, *p* = .654). The main effect of CRS was significant, *F* (1, 29) = 9.49, *p* = .004, $\eta_{{\text{p}}}^{2}$ = .25, because ERs of the CRS+ condition were higher (6.1%) than those of the CRS− condition (5.1%). The interaction of both factors was significant, *F* (3, 87) = 5.98, *p* < .001, $\eta_{{\text{p}}}^{2}$ = .17. A significant CRS effect (*p* = .024, Newman–Keuls corrected) was visible after potential no-go trials (2.4%). In contrast, the effect was not significant after go trials of the no-go task (1.9%, *p* = .139) and after go trials of the go tasks (1.2%, *p* = .230), and after no-go trials (− 1.8%, *p* = .157).

### Discussion

First, the results of Experiment II replicate the data pattern of Experiment I: Large *n* − 2 repetition costs were visible for the no-go task, while no costs occurred for the two go tasks. Regarding the main question of this experiment, the effect of task type in trial *n* suggests that participants indeed adapt their response behavior on purpose. When a no-go trial was validly excluded, responses for the no-go task were as fast as those for the go tasks. When a no-go was possible, on the contrary, responses were significantly slower. This suggests that participants prepare the task to a lower degree, or postpone full preparation to response readiness, when they expect a no-go trial to occur. However, the conclusion that effects of no-go trials are due to participants’ actively changing their response behavior does not hold for the effect of no-go trials on *n* − 2 repetition costs: All trial types of the no-go task were associated with large *n* − 2 repetition costs, while no costs occurred for the go tasks. This was even the case for trials in which a no-go trial could be validly excluded based on the cue color. This suggests that in the present study, the no-go manipulation exerted its effect on the level of task sets, not on the response level.

As in Experiment I, we then investigated the presence of competitor rule suppression. However, in contrast to Experiment I, there was no task rule congruency effect in RT data, and the effect in ER data did not show up as expected, because lowest ERs were visible for mixed congruency trials, not for fully congruent ones. Therefore, all effects with respect to competitor rule suppression should be treated with caution.^2^ This also holds for the interaction of CRS with our no-go manipulation.

A further note should be made with regard to the potential influence of cue color in this experiment. It has been shown before that cue-related processes have an influence on presence and size of *n* − 2 repetition costs. For example, Gade and Koch (2014) found *n* − 2 repetition costs to be higher for nontransparent as compared to transparent cues, an effect they relate to transparent cues leading to more distinct task representations that cause less interference and, as a consequence, less need for inhibition. In the same vein, Arbuthnott (2005) found *n* − 2 repetition benefits instead of costs when tasks were cues by unique spatial locations, a condition that is also characterized by high discriminability of the tasks. The fact that task cues were colored light grey for no-go trials and potential no-go trials (in contrast to the “usual” dark grey color for go trials) might have a comparable effect on *n* − 2 repetition costs in the present experiment. However, in this case, there should have been an interaction with the task sequence that differentially affects *n* − 2 repetition costs of no-go trials and potential no-go trials compared to the go trials of both types of tasks, irrespective of the direction of this effect. This was not the case. Therefore, although we do not state that cue-related processes have no effect on inhibitory processes in task switching, they do not affect the results of the present study.

## General discussion

The aim of the present study was to further investigate effects of no-go trials on *n* − 2 repetition costs. For this purpose, two experiments were conducted in which the occurrence of no-go trials was selectively bound to one of the tasks. Large *n* − 2 repetition costs were present when the no-go task was cued in trial *n* − 2, irrespective of whether this trial actually was a no-go trial or not. This was even the case when no-go-trials could be validly excluded in Experiment II. This suggests that the need for motor inhibition did not just add to task set inhibition, because in this case, only actual no-go trials would have been affected. These observations provide strong evidence against the assumption that response inhibition per se reduces the activation of a task to a degree that prevents inhibition to occur (as suggested by Schuch & Koch, 2003).

Instead, the pattern of results suggests that a potential need for motor inhibition is added to the task identity information in the task set of the no-go task, thereby affecting all trials with this task. Contrary to what would have been expected if the no-go task was activated to a smaller degree as compared to the other tasks, it seems that this task set, in turn, had to be inhibited to a larger degree and, therefore, caused higher *n* − 2 repetition costs.

The notion that *n* − 2 repetition costs are caused by an inhibitory process that operates at the level of task sets is in line with the work of Regev and Meiran (2016, 2017) who distinguished two independent kinds of inhibition in task switching, namely task set inhibition (as the source of *n* − 2 repetition costs) on the one hand and competitor rule suppression on the other hand. In a series of experiments, the authors showed that competitor rule suppression is affected by the ease of retrieving category–response mappings from memory, whereas *n* − 2 repetition costs are not. They conclude that *n* − 2 repetition costs are caused by an inhibition process operating at the level of abstract task identity representations, whereas competitor rule suppression targets category–response mapping rules. It is assumed that both processes are present during task switching and operate more or less independent of each other. With regard to the present results, it seems that inducing a potential need for motor inhibition selectively for one of the tasks, as it was done in the present experiments, enhances the need for subsequent task set inhibition, which then gets visible by enhanced *n* − 2 repetition costs. A possible reason for this may be that assigning no-go trials to only one of the tasks caused this task to be dominant, or more salient, in relation to the others, therefore causing asymmetric effects. With reference to the computational model of Sexton and Cooper (2017), it can be assumed that selectively assigning no-go trials to one of the tasks enhances the activation level of the task demand unit of the respective task. As a consequence, this task causes more interference and receives stronger inhibition triggered by the conflict monitoring unit. It has been shown previously that a dominant task has to be inhibited to a higher degree because it causes higher interference due to faster or higher activation (cf. Arbuthnott, 2008; Jost et al., 2017). Another possibility is that the no-go task is easily discriminated from the other two tasks based on the task cue. A similar assumption was made by Scheil and Kleinsorge (2019) who investigated effects of task repetition proportion on *n* − 2 repetition costs. They found huge costs for tasks without task repetitions, whereas there were smaller for tasks with 33% or 50% task repetitions. This was interpreted in terms of especially high utility of task inhibition tied to tasks without repetitions. The fact that the information about the utility of inhibition could be derived from the cue, because it was bound to one of the tasks, was assumed to trigger a full-blown inhibition process at an early point of time. The same kind of early onset of inhibition would have been possible in the present experiments, because the no-go condition, as the repetition proportion in Scheil and Kleinsorge’s study, could be derived from the cue. This made it possible to discriminate between the no-go task and the go tasks early in time, potentially causing full-blown task set inhibition.

One of the most peculiar findings of the present experiments consists of the observation of substantial *n* − 2 repetition costs only in those conditions that were specifically designed for the present experiments. In contrast, the ‘standard’ conditions (i.e., the go tasks) yielded either no costs or even slight *n* − 2 repetition benefits. Because the 95% confidence intervals of these effects encompassed in none of the two experiments negative values indicating *n* − 2 repetition costs, it seems to be sure that this was not a matter of statistical power (or if so, this resulted in failures to detect significant *n* − 2 repetition benefits.) This observation strongly suggests that the introduction of the no-go task resulted in more than local effects being confined to particular successions of single trials. Rather, it seems that this manipulation affected the balance of activation and inhibition across the entire ‘task space’, that is, the representation of the entire experimental situation including all potential tasks (cf. Kleinsorge et al., 2004; Xiong & Proctor, 2018). We consider this as a theoretically important point because it demonstrates that repetition benefits and costs (as well as switch costs, cf. Kleinsorge et al., 2004) cannot be attributed to isolated sequences of trials but has to take the global representation of the task environment into account. What seems to be obvious is that the introduction of no-go trials introduced some asymmetry into this global representation that endowed the corresponding task with particular salience independent of the actual processing requirements of single trials. A side effect of this seems to be that this special salience induced a particularly strong need for inhibition once this task was activated by the task cue, resulting in boosted *n* − 2 repetition costs (effect of trial *n* − 2). The flipside of this additional tonic activation of the no-go task seems to consist of a complementary lower tonic activation of the remaining tasks.

Apart from this effect on the balance of tonic activation and inhibition, the actual processing requirements in individual trials induced phasic inhibition in actual no-go trials that resulted in a slowdown of responding in trials following this no-go trial. In contrast to the *n* − 2 effect, this was not dependent on the task in trial *n* − 1 being merely associated with no-go trials but required the actual withholding of a response in trial *n* − 1. Furthermore, it seems that phasic inhibition in no-go trials was under strategic control as effects of the nature of trial *n* in Experiment 2 discriminated between trials of the no-go task that were either precued as potential no-go or as definitely go trials, a discrimination that was not observed with the effects of trial *n* − 2.

In conclusion, the present study investigated whether and how *n* − 2 repetition costs are affected by no-go trials that are bound to one of the tasks. High *n* − 2 repetition costs were visible when the no-go task was the relevant one in trial *n* − 2, irrespective of whether an actual response had to be executed or not. This indicates that the no-go manipulation exerted its effect on the level of task sets, not only on the response level. In contrast, no *n* − 2 repetition costs occurred for the tasks not associated with no-go trials, emphasizing that task sets are not represented independent of each other but are integrated into an overarching representation of the whole task environment (‘task space’). The data pattern found in the present two experiments differed from what would be expected if no-go trials occurred equally often for all tasks. It suggests that associating no-go trials to one task shifts the balance between task activation and inhibition across the whole task space.

## Footnotes

1 In addition, we tested for effects of *n* − 2 stimulus repetitions to have a more direct measure of episodic retrieval. For this purpose, we conducted an additional ANOVA with the within-subjects factors Task Sequence (CBA vs. ABA), lag2_No-Go (identity of the condition in trial *n* − 2: no-go/no-go task, go/no-go task, go/go task), and lag2_Stimulus (Repetition vs. Switch). The lag2_Stimulus factor had no effects at all, neither a main effect nor any significant interaction (all *p*’s > .188 for RTs; all *p*’s > .667 for ERs). Although the present study was not designed for investigating effects of episodic retrieval, this furthermore adds to the notion that it had no effect on the data pattern.

2 It should be noted that the present experiments were not specifically designed to investigate CRS, so these observations should not be interpreted as evidence against effects of CRS.
